# Supplementary material for: TET2 Inhibits PD-L1 Gene Expression in Breast Cancer Cells through Histone Deacetylation
Source: Cancers (Basel). 2021 May 4;13(9):2207. doi: 10.3390/cancers13092207 (PMC8125390; doi:10.3390/cancers13092207)
Supplement: Supplementary file 1 [file cancers-13-02207-s001.zip › cancers-1201219-SI.pdf]

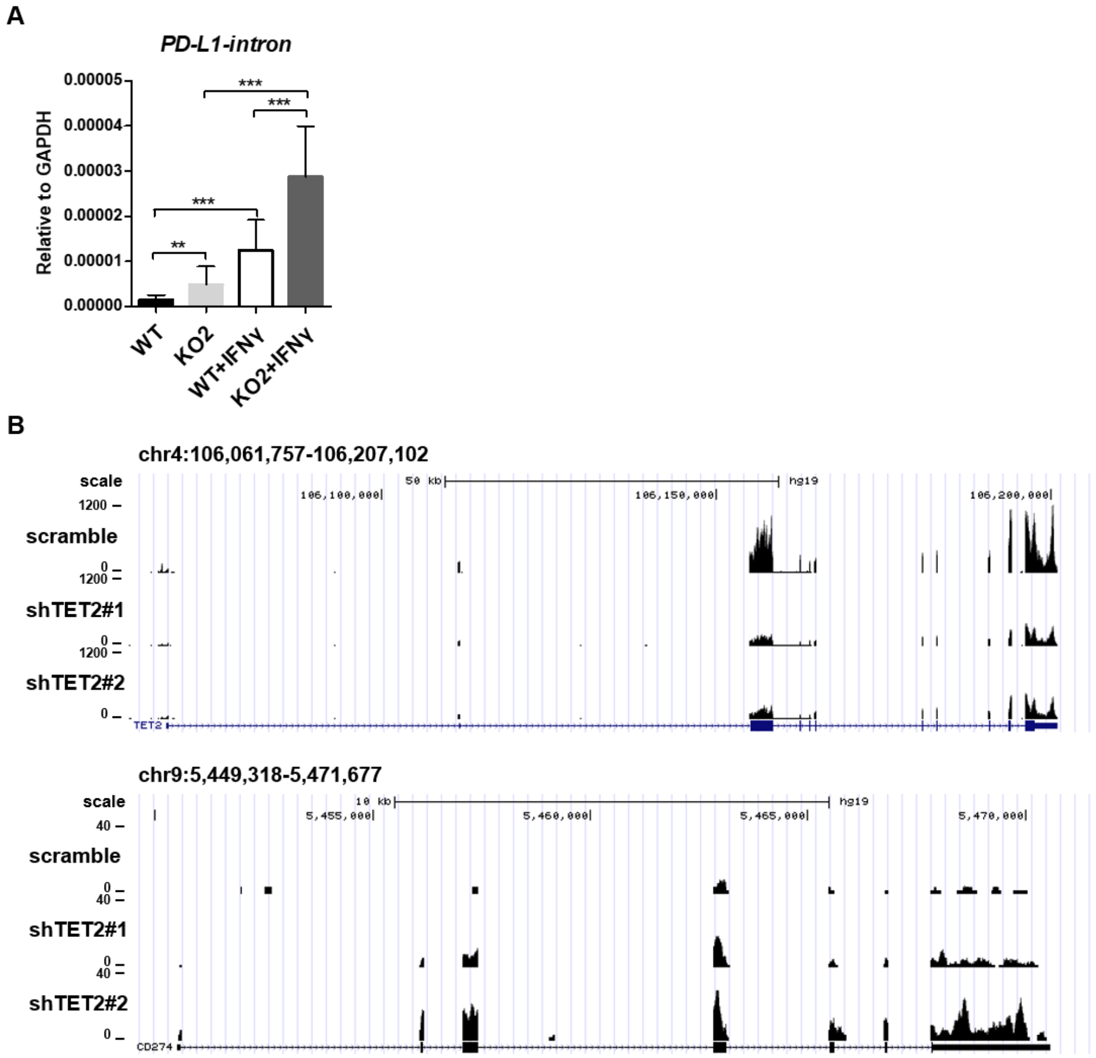

Figure S1. PD-L1 is negatively regulated by TET2 in MCF7 cells. (**A**) Relative mRNA expression of *PD-L1* intron in WT and *TET2* KO MCF7 cells treated with or without IFN-gamma. (**B**) RNA-seq snapshot of *TET2* and *PD-L1* (CD274) genes in scramble, TET2-shRNA#1, and TET2-shRNA#2 of MCF7. (***, $p < 0.001$.).

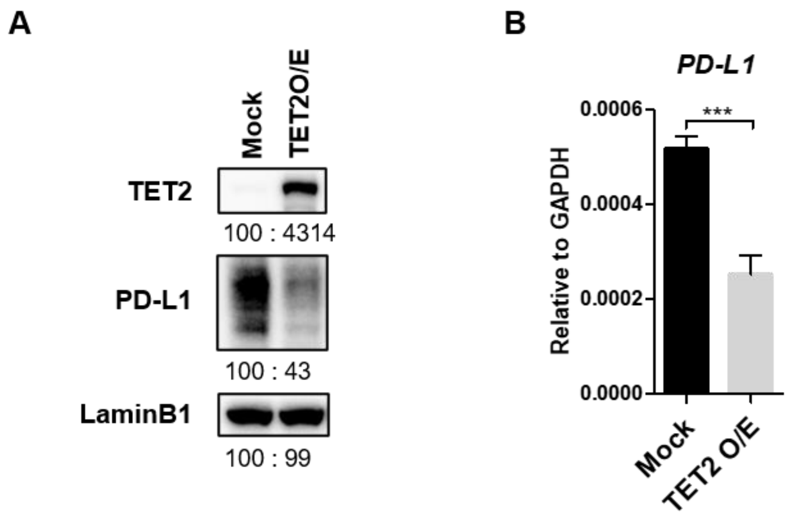

Figure S2. TET2 overexpression inhibits PD-L1 expression in MDA-MB-231 cells. (**A**) Western blot analysis of the PD-L1 protein levels in Mock and TET2-O/E of MDA-MB-231 cells. (**B**) RT-qPCR analysis of the relative mRNA expression levels of PD-L1 in Mock and TET2-O/E MDA-MB-231 cells. (***, $p < 0.001$.).

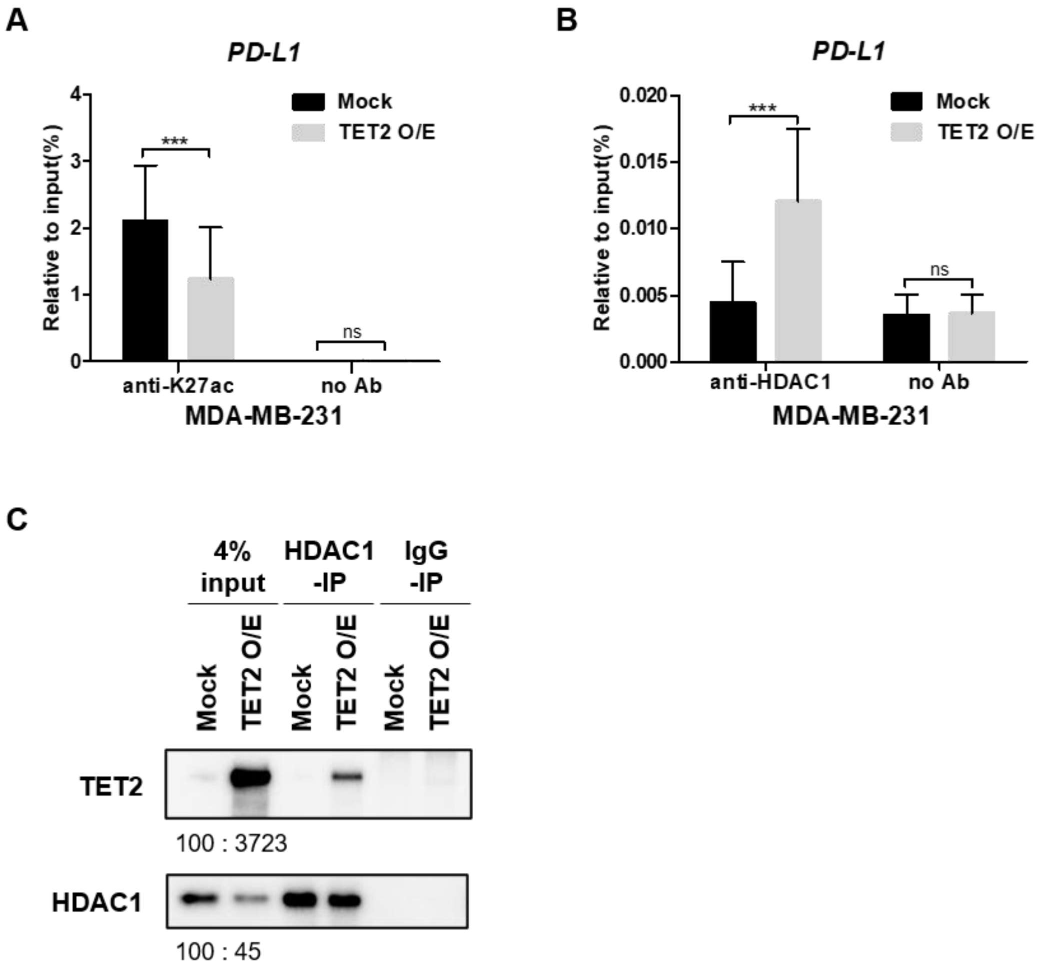

Figure S3. TET2 is required for H3K27 deacetylation and HDAC occupancy at *PD-L1* promoter in MDA-MB-231. (**A**) ChIP-qPCR analysis of H3K27ac enrichment at *PD-L1* (*CD274*) promoter in Mock and TET2-O/E MDA-MB-231 cells. (**B**) ChIP-qPCR analysis of HDAC1 occupancy at *PD-L1* (*CD274*) promoter in Mock and TET2-O/E MDA-MB-231 cells. (**C**) Western blot analysis of immunoprecipitation products with HDAC1 in Mock and TET2-O/E MDA-MB-231 cells. (***, $p < 0.001$.).

Figure 1D

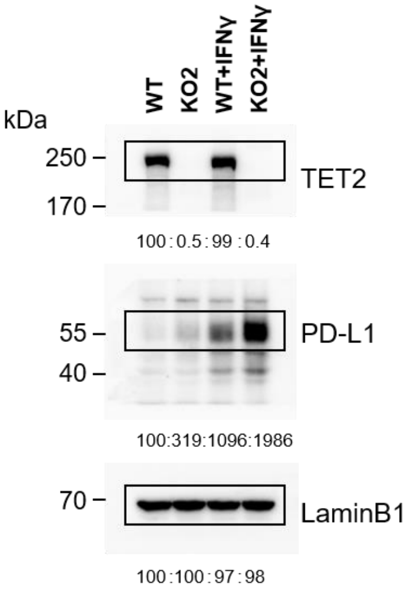

Figure 3F

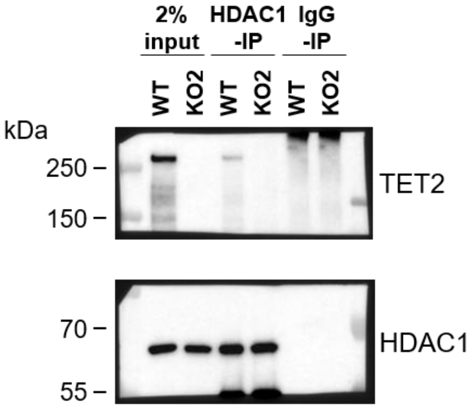

Figure 3G

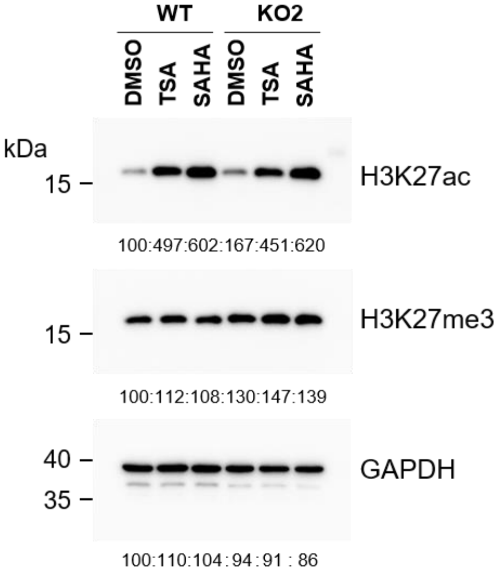

Figure S2A

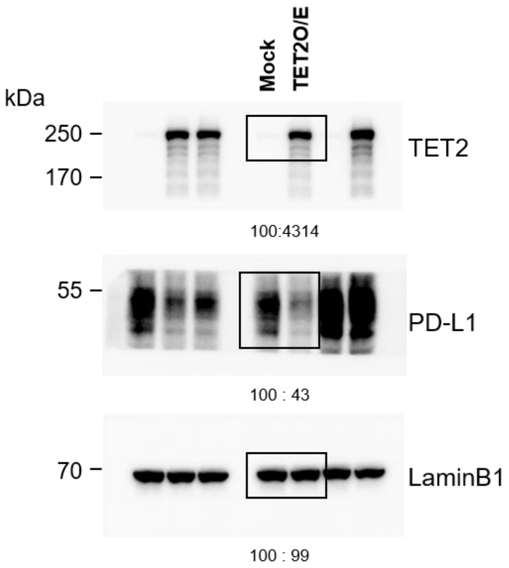

Figure S3C

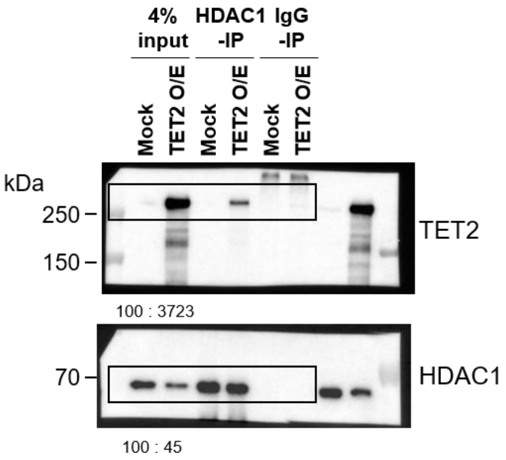

Figure S3D

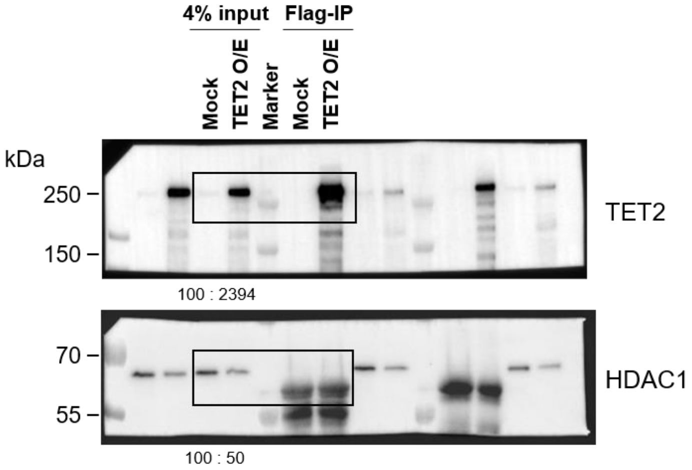

Figure S4: original Western blot figure.

Table S1. CCLE data analysis of TET2 and PD-L1 mRNA levels in most cancer cell lines.

Cancer type	n	R^2	Correlation	p value	Significance
Lung	192	0.09761	negative	< 0.0001	***
Breast	57	0.148	negative	0.0031	**
Soft tissue	31	0.2387	negative	0.0053	**
Oesophagus	27	0.1354	no	0.0589	ns
Ovary	47	0.04145	no	0.1699	ns
Pancreas	41	0.04153	no	0.2013	ns
Prostate	8	0.2133	no	0.2493	ns
Bone	28	0.05037	no	0.2509	ns
Haematopoietic and lymphoid tissue	176	0.006719	no	0.2795	ns
Central nervous system	66	0.009155	no	0.4447	ns
Kidney	32	0.01614	no	0.4884	ns
Upper aerodigestive tract	32	0.01608	no	0.4892	ns
Stomach	37	0.01324	no	0.4977	ns
Thyroid	11	0.04645	no	0.5245	ns
Urinary tract	26	0.005706	no	0.7138	ns
Pleura	11	0.009987	no	0.77	ns
Biliary tract	8	0.009115	no	0.8221	ns
Autonomic ganglia	16	0.00337	no	0.8309	ns
Skin	56	0.0005454	no	0.8643	ns
Endometrium	28	0.0009414	no	0.8768	ns
Liver	25	0.00003252	no	0.9784	ns
Large intestine	59	0.000005063	no	0.9865	ns

Table S2. CCLE data analysis of TET2 and PD-L1 expression in subtypes of breast cancer.

Breast cancer subtype	n	R^2	Correlation	p value	Significance
Luminal	20	0.2404	negative	0.0282	*
Her2-enriched	9	0.0685	no	0.4963	ns
Basal-like	28	0.1063	no	0.0904	ns

Table S3. TCGA data analysis of TET2 and PD-L1 expression in subtypes of breast cancer.

Breast cancer subtype	n	R^2	Correlation	p value	Significance
luminal B	461	0.0201	negative	0.0023	**
Her2-enriched	220	0.02008	negative	0.0357	*
luminal A	679	0.001447	no	0.3223	ns
Basal-like	199	0.0006601	no	0.7187	ns

Table S4. Primers used in the study.

Gene name	Primers sequence	Notes
**RT-qPCR primers**		
GAPDH_F	CTGACTTCAACAGCGACACC	
GAPDH_R	GTGGTCCAGGGGTCTTACTC	
PD-L1_F	TCTGGACAAGCAGTGACCATC	
PD-L1_R	CAGTGTGCTGGTCACATTGAA	
PD-L1-intron_F	CCCCAACCTGAATGAGCCAT	
PD-L1-intron_R	GTGAGGGACGAAAAGGCAGA	
TET2_F	CCACCTCATCTCAAGCTGCA	
TET2_R	GATCCATCGGCTGAGACTGG	
**ChIP-qPCR, MeDIP/hMeDIP-qPCR primers**		
CD274p_F	AACTGAAAGCTTCCGCCGATT	For ChIP of TET2,HDAC1/2,H3K4me3 and H3K27me3; for MeDIP/hMeDIP
CD274p_R	ATCCAGTTTGCCGGGCG	
K27ac-CD274p-F	GGGGGACGCCTTTCTGATAA	For ChIP of H3K27ac
K27ac-CD274p-R	AAGCCAACATCTGAACGCAC	
